# Association of Interleukin-18 Gene Promoter −607 C>A and −137G>C Polymorphisms with Cancer Risk: A Meta-Analysis of 26 Studies

**DOI:** 10.1371/journal.pone.0073671

**Published:** 2013-09-16

**Authors:** Xin Yang, Man-Tang Qiu, Jing-Wen Hu, Feng Jiang, Ming Li, Jie Wang, Qin Zhang, Rong Yin, Lin Xu

**Affiliations:** 1 The First Clinical College of Nanjing Medical University, Nanjing, China; 2 Department of Thoracic Surgery, Nanjing Medical University Affiliated Cancer Hospital, Cancer Institute of Jiangsu Province, Nanjing, China; 3 The Fourth Clinical College of Nanjing Medical University, Nanjing, China; 4 Department of Scientific Research, Nanjing Medical University Affiliated Cancer Hospital, Cancer Institute of Jiangsu Province, Nanjing, China; Faculdade de Medicina, Universidade de São Paulo, Brazil

## Abstract

**Background:**

Evidence suggest that IL-18 gene polymorphisms may be risk factors for several cancers. Increasing studies investigating the association between IL-18 gene promoter polymorphisms (−607 C>A and −137G>C) and cancer risk have yielded conflicting results.

**Methodology/Principal Findings:**

We performed a meta-analysis of 26 studies including 4096 cases and 5222 controls. We assessed the strength of the association of IL-18 gene promoter −607 C>A and −137G>C polymorphisms with cancer risk and performed sub-group analyses by cancer types, ethnicities, source of controls and sample size. The pooled results revealed a significant increased risk of cancer susceptibility for −607 C>A (CA vs. CC: OR = 1.19, 95%CI: 1.04, 1.37, P_heterogeneity_ = 0.033; CA/AA vs. CC: OR = 1.17, 95% CI: 1.01, 1.34, P_heterogeneity_ = 0.007), but no significant association for −137 G>C was observed with overall cancer risk. Sub-group analyses revealed that an increased risk of nasopharyngeal carcinoma was both found for −607 C>A (CA/AA vs. CC: OR = 1.32, 95% CI: 1.04, 1.69, P_heterogeneity_ = 0.823) and −137G>C (GC/CC vs. GG: OR = 1.57, 95%CI: 1.26, 1.96, P_heterogeneity_ = 0.373). Consistent with the results of the genotyping analyses, the −607A/−137C and −607C/−137C haplotypes were associated with a significantly increased risk of nasopharyngeal carcinoma as compared with the −607C/−137G haplotype (−607A/−137C vs. −607C/−137G: OR = 1.26, 95%CI: 1.13, 1.40; P_heterogeneity_ = 0.569; −607C/−137C vs. −607C/−137G: OR = 1.14, 95%CI: 1.03, 1.27; P_heterogeneity_ = 0.775). As for gastrointestinal cancer, we also found that −607 C>A polymorphism was significantly associated with increased cancer risk (CA/AA vs. CC: OR = 1.25, 95% CI: 1.05, 1.50, P_heterogeneity_ = 0.458). Further sub-group analysis revealed that −137G>C polymorphism contributed to cancer risk in Asians but not in Caucasians (GC/CC vs. GG: OR = 1.31, 95%CI: 1.05, 1.64, P_heterogeneity_<0.001).

**Conclusions:**

The meta-analysis results suggest that IL-18 gene promoter −607 C>A polymorphism is significantly associated with overall cancer risk, especially in nasopharyngeal carcinoma and gastrointestinal cancer; and the −137 G>C polymorphism is associated with increased overall cancer risk in Asian populations and also significantly increases the risk of nasopharyngeal carcinoma.

## Introduction

Interleukin-18 (IL-18) is a member of the IL-1 cytokine family, and it is initially described as IFN-γ inducing factor [1]. IL-18 is produced by various cells, including T and B cells, and a range of antigen-presenting cells including activated monocytes, dendritic cells and macrophages, which can regulate both innate and adaptive immune responses [2], [3]. Evidence has indicated that IL-18 might possess anticancer function. IL-18 can stimulate natural killer cells and T cells promoting primarily Th1 response, which is able to increase the immune defense against tumor cells by activating and inducing the production of IFN-γ [4]. The mechanisms of the host defense against cancer are very complex, including suppression of tumor growth [5], induction of cancer cell apoptosis [6], and inhibition of angiogenesis [7]. However, IL-18 has also been found to promote tumor progression. Higher expression of IL-18 is detected in various cancer cells compared with normal control, and IL-18 is able to induce angiogenesis, migration, proliferation and immune escape [8]. These findings confirm the evidence of an association between IL-18 gene and cancer risk but remain controversial.

The IL-18 gene is located on chromosome 11q22.2–q22.3, and contains many polymorphisms, especially in the promoter region. The variations in IL-18 gene promoter are able to influence IL-18 production and activity. The IL-18 gene promoter −607 C>A (rs1946518) and −137 G>C (rs187238) polymorphisms are two of the most common single nucleotide polymorphisms (SNPs). The −607 C>A can alter a cAMP-responsible element binding site, and result in a decrease of IL-18 transcription [9]. The −137 G>C can change the binding site of histone 4 transcription factor-1(H4TF-1) nuclear factor. Additionally, cloning and gene expression analysis showed that the polymorphisms in IL-18 promoter region caused the differences in transcription factor binding and had an impact on IL-18 gene activity [9]. Recently, The IL-18 gene polymorphisms have been investigated in several cancers such as nasopharyngeal carcinoma [10], [11], prostate cancer [12], colorectal cancer [13], esophageal carcinoma [14], cervical cancer [15], breast cancer [16] and so on. However, these studies yielded different or even controversial results.

Meta-analysis is a means of increasing the effective sample size under investigation through the pooling of data from individual studies, thus enhancing the statistical power of the analysis for the estimation of genetic effects [17]. To clarify the association between IL-18 gene promoter polymorphisms and cancer risk, we performed this meta-analysis by pooling eligible studies to calculate the estimate of overall cancer risk and evaluated influence of cancer types, ethnicity, source of controls and sample size.

## Methods

### Search Strategy

According to the Preferred Reporting Items for Systematic Reviews and Meta-Analyses (PRISMA), we conducted a systematic literature search using the databases PubMed, EMBASE and CNKI (Chinese National Knowledge Infrastructure) without language, time period and sample size limitations, covering all papers published up to April 10, 2013, with a combination of the following key words: IL-18 gene (e.g.: “IL-18”, and “Interleukin-18”); cancer (e.g.: “cancer”, “carcinoma”, “tumor” or “neoplasms”) and polymorphism or variation. Furthermore, all searched papers including reviews were retrieved, and their references were checked as well for other relevant publications.

### Inclusion and Exclusion Criteria

The following criteria were used for the literature selection: (a) only the case–control studies were considered; (b) the association of cancer risk with −607 C>A and −137 G>C polymorphisms was clearly investigated; (c) sufficient genotype distribution information in cases and controls. The major reasons for exclusion of studies were (a) reviews and repeated literature; (b) study design other than case-control method; (c) studies without detailed genotype frequencies.

### Data Extraction

The following information was independently extracted from each study by two authors (Yang and Qiu) according to the selection criteria mentioned above: name of first author, publication year, country where the study was conducted, ethnicity, source of controls, cancer types, genotyping methods, genotype frequency in cases and controls. Different ethnicities were categorized as Asian, Caucasian, and African. Cancer types were classified as Gynecological cancer (GC), including cervical cancer, ovarian cancer, choriocarcinoma; Genitourinary system cancer (GUC), including prostate cancer, renal cell carcinoma and bladder cancer; Gastrointestinal cancer (GIC), including esophageal carcinoma, stomach cancer and colorectal cancer; Nasopharyngeal carcinoma (NC); Breast cancer (BC) and Others (oral cancer, head and neck carcinoma, lung cancer). All eligible studies were defined as hospital-based (HB) and population-based (PB) according to the source of controls. The Hardy–Weinberg equilibrium (HWE) were calculated by Chi-square test (p<0.05 was considered as significant disequilibrium) based on the two polymorphisms genotyping distribution in controls [18].

### Statistical Analysis

Odds ratio (OR) with 95% confidence intervals (CIs) was used to assess the strength of association between IL-18 gene promoter polymorphisms (−607 C>A and −137G>C) and cancer risk, based on the genotype frequencies in cases and controls. A 95% CI was used for statistical significance test and it without 1 for OR indicating a significant increased or reduced cancer risk. The pooled ORs were calculated for four models respectively: homozygote comparison (AA vs. CC; CC vs. GG), heterozygote comparison (CA vs. CC; GC vs. GG), dominant model (CA/AA vs. CC; GC/CC vs. GG) and recessive model (AA vs. CC/CA; CC vs. GG/GC). The haplotypes were divided into four categories: −607A/−137C, −607A/−137G, −607C/−137C and −607C/−137G. Fixed-effects model (Mantel-Haenszel method) was adopted when P_heterogeneity_ was more than 0.10, while random-effects model (the Der Simonian and Laird method) was more appropriate when P_heterogeneity_ was less than 0.10 [19], [20]. Sensitivity analysis was conducted by removing one data set at a time to identify individual study’ effect on pooled results and test the reliability of results [18]. The heterogeneity between these studies was checked using Chi-square based Q test and it was considered statistically significant when P-value was less than 0.10. Sub-group analyses and logistic meta-regression analyses were conducted to explore the source of heterogeneity among variables, such as years, cancer types, ethnicities, source of controls and sample size (studies with more than 500 participants were defined as “large”, and studies with less 500 participants were defined as “small”). Begg’s funnel plots [21] and Egger’s regression method [22] were conducted to detect the potential publication bias (P<0.05 was considered representative of statistically significant publication bias). All P values are two-sided. Statistical analysis was done using STATA software (version 12.1; Stata Corp, College Station, Texas USA).

## Results

### Characteristics of Studies

**Figure 1:**
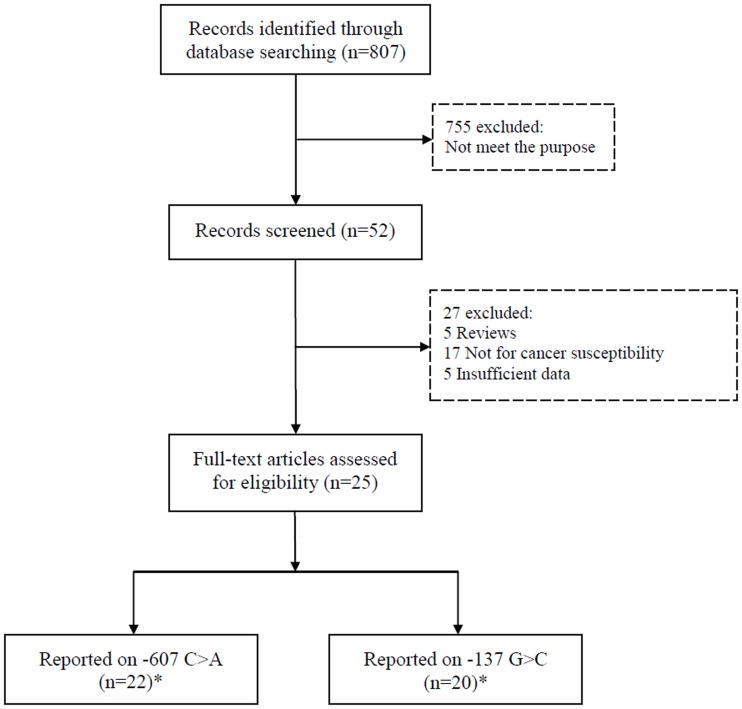
PRISMA Flow Chart. *Two separate studies were reported in one article, thus 23 studies on −607 C>A and 21 studies on −137 G>C were eligible.

The detailed study selection process was shown in Figure 1. In the study reported by Haghshenas and colleagues, the cancer types contained colorectal and stomach cancer, and the genotype frequencies were presented separately, thus each of them was considered as a separate study in this meta-analysis. A total of 23 studies for −607 C>A and 21 studies for −137G>C were finally included with 4096 cases and 5222 controls according to selection criteria[10]–[16], [23]–[40]. The detailed characteristics of the eligible studies included in this meta-analysis are shown in Table 1.

**Table 1:** Characteristics of Eligible Studies.

Firstauthor	Year	Ethnicity	Control	Cancer types	Genotypingmethod	−607 C>A							−137 G>C						
						Cases			Controls			HWE	Cases			Controls			HWE
						CC	CA	AA	CC	CA	AA		GG	GC	CC	GG	GC	CC	
**Bushley**	2004	Mixed	PB	ovarian cancer	Taqman	NA	NA	NA	NA	NA	NA	NA	127	48	7	139	71	9	0.99
**Pratesi**	2006	Caucasian	PB	nasopharyngealcarcinoma	AS-PCR	26	42	21	43	64	23	0.92	43	39	7	72	53	5	0.21
**Liu**	2007	Asian	HB	prostate cancer	AS-PCR	50	143	72	65	137	78	0.75	149	96	20	195	73	12	0.13
**Nikiteas**	2007	Caucasian	PB	colorectal cancer	PCR-RFLP	19	47	18	35	32	22	0.01	NA	NA	NA	NA	NA	NA	NA
**Wei**	2007	Asian	HB	esophagealsquamous cellcarcinoma	AS-PCR	48	123	64	59	124	67	0.91	127	91	17	176	66	8	0.56
**Vairaktaris**	2007	Caucasian	PB	oral cancer	PCR-RFLP	55	66	28	35	32	22	0.01	NA	NA	NA	NA	NA	NA	NA
**Yang**	2007	Asian	PB	cervical cancer	Taqman	33	50	24	18	26	36	<0.01	NA	NA	NA	NA	NA	NA	NA
**Sobti**	2008	Asian	HB	cervical cancer	PCR-SSP	NA	NA	NA	NA	NA	NA	NA	89	104	7	114	74	12	1.00
**Farhat**	2008	African	PB	nasopharyngealcarcinoma	PCR-RFLP	41	94	28	53	77	34	0.54	75	73	15	83	68	13	0.86
**Kashef**	2008	Asian	PB	choriocarcinoma	AS-PCR	6	10	3	33	54	16	0.43	8	8	3	56	39	8	0.74
**Qi**	2008	Asian	HB	cervical cancer	AS-PCR	5	17	28	17	24	9	0.92	NA	NA	NA	NA	NA	NA	NA
**Khalili**	2009	Asian	PB	breast cancer	AS-PCR	64	103	33	76	97	33	0.83	141	96	13	110	72	24	0.03
**Asefi**	2009	Asian	HB	head and necksquamous cellcarcinoma	AS-PCR	43	53	15	82	101	29	0.81	65	37	9	116	79	17	0.50
**Haghshenas**	2009	Asian	PB	stomach cancer	AS-PCR	31	40	16	119	144	48	0.68	56	28	4	167	109	33	0.02
**Haghshenas**	2009	Asian	PB	colorectal cancer	AS-PCR	55	72	15	119	144	48	0.68	83	55	5	167	109	33	0.02
**Samsami**	2009	Asian	HB	ovarian cancer	AS-PCR	22	51	12	57	75	26	0.87	46	34	5	81	57	20	0.06
**Nong**	2009	Asian	PB	nasopharyngealcarcinoma	PCR-RFLP	47	132	71	69	133	68	0.81	140	88	22	189	70	11	0.17
**Farjadfar**	2009	Asian	HB	lung cancer	AS-PCR	15	45	13	40	46	11	0.68	33	33	7	53	35	9	0.37
**Saenz**	2010	Caucasian	PB	renal cellcarcinoma	Taqman	59	76	19	166	261	73	0.07	91	59	6	251	220	31	0.06
**Monroy**	2011	Caucasian	PB	hodgkin disease	MassARRAY	NA	NA	NA	NA	NA	NA	NA	85	9	7	53	42	5	0.36
**Taheri**	2012	Asian	PB	breast cancer	T-ARMS	29	32	11	40	45	8	0.35	NA	NA	NA	NA	NA	NA	NA
**Babar**	2012	Caucasian	PB	esophagealadenocarcinoma	Taqman	384	508	178	83	75	36	0.01	105	74	14	582	414	86	0.31
**Guo**	2012	Asian	HB	colorectal cancer	PCR-RFLP	36	85	49	42	76	42	0.53	91	65	14	112	41	7	0.21
**Du**	2012	Asian	HB	nasopharyngealcarcinoma	PCR-RFLP	36	80	34	47	93	40	0.64	88	51	11	131	43	6	0.30
**Jaiswal**	2013	Asian	HB	bladder cancer	PCR-RFLP	81	89	30	61	113	36	0.18	82	112	6	118	77	5	0.06
**Liu**	2013	Asian	PB	prostate cancer	AS-PCR	100	172	103	94	196	110	0.71	301	74	0	304	94	2	0.06

PB: population-based; HB: hospital-based; HWE: Hardy–Weinberg equilibrium.

### Association of −607 C>A with Cancers Risk

**Figure 2:**
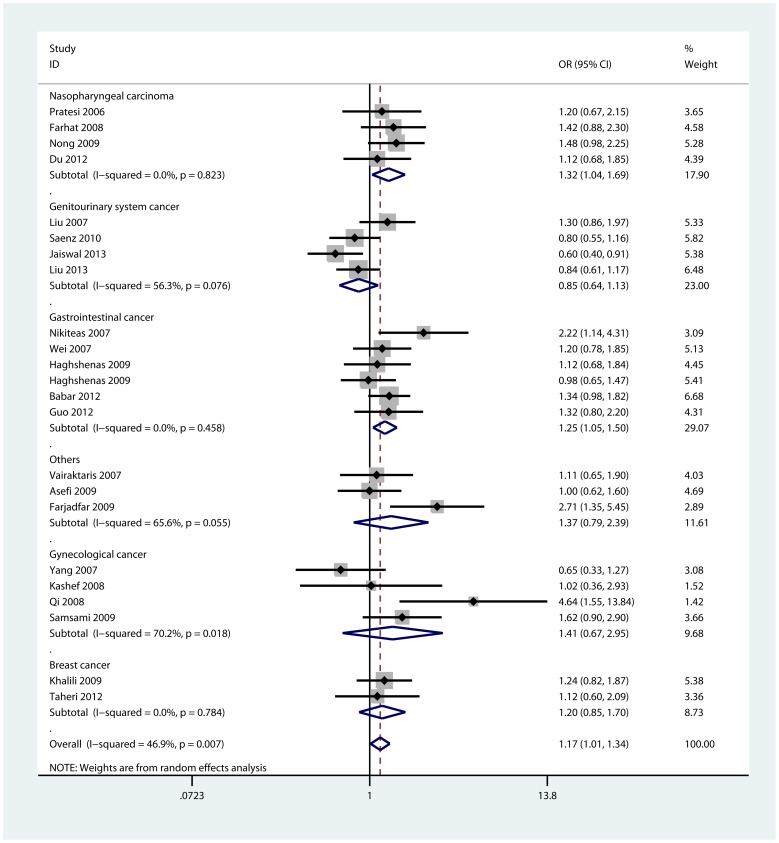
Forest plot of −607 C>A dominant model for overall comparison by cancer types (CA/AA vs. CC).

As shown in Table 2, we observed a significant increased risk of cancer susceptibility in heterozygote comparison (CA vs. CC: OR = 1.19, 95%CI: 1.04, 1.37; P_heterogeneity_ = 0.033) and dominant model (CA/AA vs. CC: OR = 1.17, 95% CI: 1.01, 1.34; P_heterogeneity_ = 0.007, Figure 2) when all eligible studies were pooled. However, we found no significant association in homozygote comparison (AA vs. CC: OR = 1.11, 95%CI: 0.92, 1.33; P_heterogeneity_ = 0.013) or recessive model (AA vs. CC/CA: OR = 0.99, 95% CI: 0.85, 1.15; P_heterogeneity_ = 0.032).

**Table 2:** Results from meta-analysis of −607 C>A and cancer risk.

		AA vs. CC		CA vs. CC			CA/AA vs. CC		AA vs. CC/CA	
	N	OR	P_h_	OR		P_h_	OR	P_h_	OR	P_h_
**Total**	23	1.11(0.92, 1.33)	0.013	1.19(1.04, 1.37)*		0.033	1.17(1.01, 1.34)*	0.007	0.99(0.85, 1.15)	0.032
**Cancer Types**										
**GC**	4	1.41(0.36, 5.45)	<0.001	1.45(0.97, 2.18)		0.525	1.41(0.67, 2.95)	0.018	1.12(0.33, 3.81)	<0.001
**NC**	4	1.31(0.96, 1.77)	0.759	1.33(1.03, 1.72)*		0.704	1.32(1.04, 1.69)*	0.823	1.08(0.84, 1.39)	0.547
**GUC**	4	0.87(0.67, 1.12)	0.363	0.86(0.63, 1.16)		0.068	0.85(0.64, 1.13)	0.076	0.94(0.77, 1.15)	0.914
**GIC**	6	1.12(0.88, 1.42)	0.648	1.32(1.08, 1.63)*		0.327	1.25(1.05, 1.50)*	0.458	0.95(0.78, 1.17)	0.681
**BC**	2	1.33(0.80, 2.22)	0.438	1.17(0.81, 1.68)		0.532	1.20(0.85, 1.70)	0.784	1.23(0.72, 2.10)	0.274
**Others**	3	1.26(0.61, 2.62)	0.080	1.43(0.85, 2.42)		0.100	1.37(0.79, 2.39)	0.055	0.98(0.62, 1.56)	0.277
**Ethnicities**										
**Asian**	17	1.15(0.90, 1.46)	0.003	1.15(0.98, 1.34)	0.099		1.15(0.97, 1.37)	0.010	1.04(0.85, 1.25)	0.011
**Caucasian**	5	1.02(0.78, 1.34)	0.473	1.29(0.91, 1.85)	0.041		1.20(0.89, 1.62)	0.083	0.89(0.70, 1.14)	0.620
**African**	1	1.06(0.56, 2.03)	N/A	1.58(0.95, 2.62)	N/A		1.42(0.88, 2.30)	N/A	0.79(0.46, 1.38)	N/A
**Source of Controls**										
**PB**	14	1.01(0.83, 1.23)	0.196	1.18(1.01, 1.37)*	0.189		1.12(0.97, 1.29)	0.190	0.91(0.76, 1.09)	0.154
**HB**	9	1.33(0.92, 1.91)	0.009	1.25(0.94, 1.65)	0.018		1.30(0.96, 1.75)	0.002	1.14(0.87, 1.48)	0.044
**Sample Size**										
**Large^a^**	4	1.05(0.77, 1.42)	0.192	1.05(0.78, 1.42)	0.080		1.05(0.78, 1.41)	0.063	1.01(0.83, 1.22)	0.750
**Small^b^**	19	1.14(0.91, 1.42)	0.011	1.24(1.06, 1.45)*	0.086		1.21(1.03, 1.42)*	0.019	0.99(0.82, 1.21)	0.011

GC: Gynecological cancer; NC: Nasopharyngeal carcinoma; GUC: Genitourinary system cancer; GIC: Gastrointestinal cancer; BC: Breast cancer; N: number of studies included; OR: odds ratio; P_h_: p value for heterogeneity;

* OR with statistical significance; a: studies with more than 500 participants; b: studies with less than 500 participants;

In the stratified analyses by cancer types, increased cancer risk was found in heterozygote comparison (CA vs. CC: OR = 1.33, 95%CI: 1.03, 1.72; P_heterogeneity_ = 0.704) and dominant model (CA/AA vs. CC: OR = 1.32, 95% CI: 1.04, 1.69; P_heterogeneity_ = 0.823, Figure 2) for nasopharyngeal carcinoma. As for gastrointestinal cancer, we also found that the −607 C>A polymorphism was significantly associated with increased cancer risk in heterozygote comparison (CA vs. CC: OR = 1.32, 95%CI: 1.08, 1.63; P_heterogeneity_ = 0.327) and dominant model (CA/AA vs. CC: OR = 1.25, 95% CI: 1.05, 1.50; P_heterogeneity_ = 0.458, Figure 2). However, no significant association was observed for other cancer types (Table 2). It’s worth noting that a trend of decreased risk could be drawn only in genitourinary system cancer. When stratified by source of controls, we only found a significant increased risk of cancer susceptibility in population-based studies (CA vs. CC: OR = 1.18, 95%CI: 1.01, 1.37; P_heterogeneity_ = 0.189, Figure S1). In terms of sub-group analyses by sample size, the associations were significant in studies with small sample size among two models: heterozygote comparison (CA vs. CC: OR = 1.24, 95%CI: 1.06, 1.45; P_heterogeneity_ = 0.086) and dominant model (CA/AA vs. CC: OR = 1.21, 95% CI: 1.03, 1.42; P_heterogeneity_ = 0.019, Figure S2). Further analyses did not show any associations between −607 C>A polymorphism and cancer risk in different ethnicities.

### Association of −137 G>C with Cancers Risk

As shown in Table 3, we found no significant association of the −137 G>C polymorphism in IL-18 promoter region with overall cancer risk in any of four models.

**Table 3:** Results from meta-analysis of −137 G>C and cancer risk.

		CC vs. GG		GC vs. GG			GC/CC vs. GG		CC vs. GG/GC	
	N	OR	P_h_	OR		P_h_	OR	P_h_	OR	P_h_
**Total**	21	1.09(0.78, 1.51)	<0.001	1.15(0.94, 1.40)		<0.001	1.13(0.92, 1.39)	<0.001	1.02(0.76, 1.37)	0.005
**Cancer Types**										
**GC**	4	0.80(0.44, 1.47)	0.301	1.17(0.73, 1.87)		0.032	1.11(0.72, 1.73)	0.037	0.75(0.41, 1.38)	0.274
**NC**	4	2.10(1.34, 3.29)*	0.538	1.48(1.18, 1.86)*		0.512	1.57(1.26, 1.96)*	0.373	1.82(1.17, 2.84)*	0.611
**GUC**	4	1.10(0.45, 2.72)	0.065	1.20(0.72, 2.00)		<0.001	1.19(0.70, 2.04)	<0.001	1.04(0.52, 2.09)	0.197
**GIC**	5	0.96(0.41, 2.23)	0.001	1.24(0.87, 1.76)		0.007	1.18(0.78, 1.80)	<0.001	0.90(0.43, 1.88)	0.005
**Others**	4	0.73(0.44, 1.21)	0.310	0.68(0.30, 1.51)		<0.001	0.71(0.37, 1.36)	<0.001	0.80(0.45, 1.41)	0.197
**Ethnicities**										
**Asian**	15	1.13(0.72, 1.78)	<0.001	1.35(1.12, 1.64)*	0.001		1.31(1.05, 1.64)*	<0.001	1.00(0.66, 1.51)	0.001
**Caucasian**	4	0.91(0.55, 1.51)	0.296	0.64(0.33, 1.23)	<0.001		0.70(0.39, 1.24)	<0.001	0.99(0.63, 1.54)	0.360
**African**	1	1.28(0.57, 2.86)	N/A	1.19(0.75, 1.87)	N/A		1.20(0.78, 1.86)	N/A	1.18(0.54, 2.56)	N/A
**Mixed**	1	0.85(0.31, 2.35)	N/A	0.74(0.48, 1.15)	N/A		0.75(0.49, 1.14)	N/A	0.93(0.34, 2.56)	N/A
**Source of Controls**										
**PB**	12	0.85(0.55, 1.33)	0.005	0.90(0.70, 1.15)	<0.001		0.88(0.69, 1.13)	<0.001	0.87(0.58, 1.31)	0.013
**HB**	9	1.49(0.97, 2.27)	0.078	1.62(1.34, 1.96)*	0.131		1.59(1.29, 1.97)*	0.033	1.26(0.85, 1.86)	0.129
**Sample Size**										
**Large** a	4	1.30(0.51, 3.27)	0.019	1.14(0.73, 1.80)	<0.001		1.16(0.70, 1.92)	<0.001	1.27(0.60, 2.70)	0.073
**Small** b	17	1.03(0.73, 1.46)	0.003	1.14(0.91, 1.45)	<0.001		1.12(0.89, 1.42)	<0.001	0.97(0.70, 1.33)	0.016

**Figure 3:**
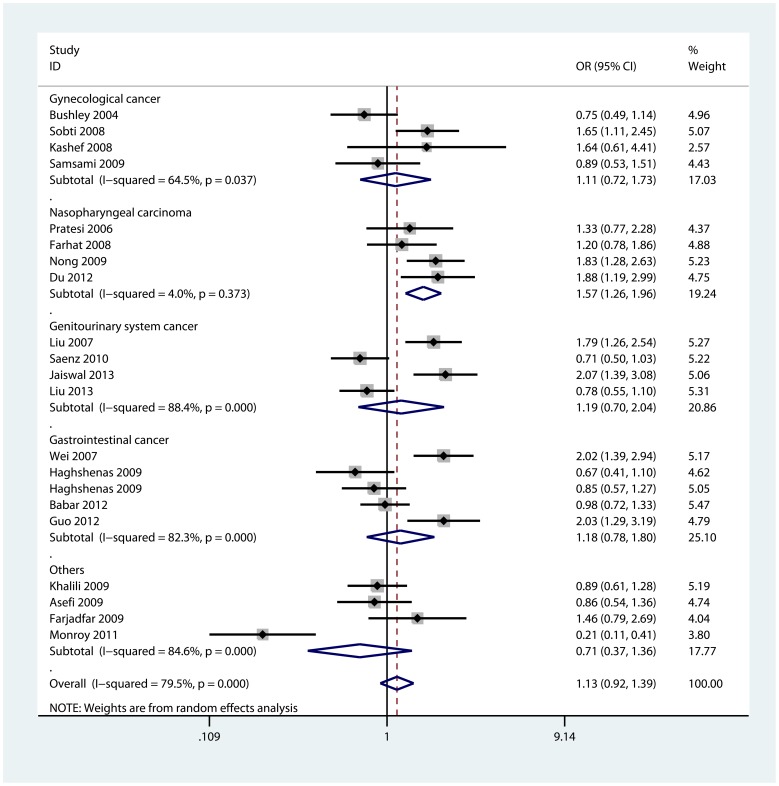
Forest plot of −137 G>C dominant model for overall comparison by cancer types (GC/CC vs. GG).

GC: Gynecological cancer; NC: Nasopharyngeal carcinoma; GUC: Genitourinary system cancer; GIC: Gastrointestinal cancer; N: number of studies included; OR: odds ratio; P_h_: p value for heterogeneity;

* OR with statistical significance;

a studies with more than 500 participants;

b studies with less than 500 participants;

When stratified by cancer types, it was found that individuals with the C allele had higher risk of nasopharyngeal carcinoma in four models : homozygote comparison (CC vs. GG: OR = 2.10, 95%CI: 1.34, 3.29; P_heterogeneity_ = 0.538), heterozygote comparison (GC vs. GG: OR = 1.48, 95%CI: 1.18, 1.86; P_heterogeneity_ = 0.512), dominant model (GC/CC vs. GG: OR = 1.57, 95%CI: 1.26, 1.96; P_heterogeneity_ = 0.373, Figure 3), and recessive model (CC vs. GG/GC: OR = 1.82, 95%CI: 1.17, 2.84; P_heterogeneity_ = 0.611). However, no significant association was observed for other cancer types (Table 3). In the stratified analyses by ethnicities, the association were only significant in Asian populations for two models: heterozygote comparison (GC vs. GG: OR = 1.35, 95%CI: 1.12, 1.64; P_heterogeneity_ = 0.001), and dominant model (GC/CC vs. GG: OR = 1.31, 95%CI: 1.05, 1.64; P_heterogeneity_<0.001, Figure S3). In terms of sub-group analyses by the source of controls, we only found significant increased risk of cancer in hospital-based studies for two models: heterozygote comparison (GC vs. GG: OR = 1.62, 95%CI: 1.34, 1.96; P_heterogeneity_ = 0.131), and dominant model (GC/CC vs. GG: OR = 1.59, 95%CI: 1.29, 1.97; P_heterogeneity_ = 0.033, Figure S4). Further analyses showed no significant results in population-based studies and studies of different sample size.

### IL-18 Gene Promoter Haplotypes and Cancer Risk

IL-18 promoter −607 C>A and −137 G>C polymorphisms showed strong linkage disequilibrium [10], [12], [14], [28], which was also confirmed by HaploView software (version 4.2). In overall analysis, no haplotype was correlated with a significantly increased risk of overall cancers (Table 4). However, when stratified by haplotypes, we found
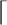
−607A/−137C and −607C/−137C haplotypes were associated with a significantly increased risk of nasopharyngeal carcinoma as compared with the −607C/−137G
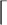
haplotype (−607A/−137C vs. −607C/−137G: OR = 1.26, 95%CI: 1.13, 1.40; P_heterogeneity_ = 0.569; −607C/−137C vs. −607C/−137G: OR = 1.14, 95%CI: 1.03, 1.27; P_heterogeneity_ = 0.775; Table 4).

**Table 4:** Results from meta-analysis of IL-18 gene promoter haplotypes.

		−607A/−137C vs. −607C/−137G		−607A/−137G vs. −607C/−137G		−607C/−137C vs. −607C/−137G	
	N	OR	P_h_	OR	P_h_	OR	P_h_
**Total**	26	1.08(0.97, 1.21)	<0.001	1.01(0.95, 1.08)	<0.001	1.04(0.93, 1.17)	<0.001
**Cancer Types**							
**GC**	6	1.13(0.76, 1.68)	<0.001	1.07(0.79, 1.45)	<0.001	1.00(0.86, 1.17)	0.389
**NC**	4	1.26(1.13, 1.40)*	0.569	1.05(0.96, 1.16)	0.951	1.14(1.03, 1.27)*	0.775
**GUC**	4	0.99(0.83, 1.19)	0.008	0.97(0.90, 1.04)	0.631	1.06(0.92, 1.21)	0.051
**GIC**	6	1.17(0.94, 1.47)	<0.001	0.99(0.85, 1.14)	0.001	1.16(0.84, 1.59)	<0.001
**BC**	2	0.98(0.76, 1.27)	0.242	1.09(0.93, 1.27)	0.570	0.88(0.74, 1.04)	0.454
**Others**	4	0.87(0.56, 1.37)	<0.001	1.05(0.91, 1.20)	0.537	0.83(0.60, 1.17)	0.006
**Ethnicities**							
**Asian**	18	1.12(0.99, 1.26)	<0.001	1.03(0.96, 1.11)	0.006	1.06(0.98, 1.14)	0.044
**Caucasian**	6	0.98(0.69, 1.38)	<0.001	0.95(0.82, 1.11)	0.018	0.98(0.64, 1.49)	<0.001
**African**	1	1.11(0.88, 1.38)	NA	1.03(0.84, 1.25)	NA	1.05(0.85, 1.31)	NA
**Mixed**	1	0.81(0.56, 1.15)	NA	1.00(0.80, 1.24)	NA	0.81(0.56, 1.15)	NA
**Source of Controls**							
**PB**	16	0.97(0.83, 1.14)	<0.001	0.98(0.90,1.06)	0.003	0.98(0.82, 1.18)	<0.001
**HB**	10	1.25(1.10, 1.42)*	0.003	1.06(0.96, 1.18)	0.006	1.15(1.07, 1.23)*	0.487
**Sample Size**							
**Large** a	4	1.06(0.84, 1.34)	<0.001	1.01(0.94, 1.08)	0.627	1.05(0.92, 1.21)	0.056
**Small** b	22	1.09(0.96, 1.24)	<0.001	1.02(0.94, 1.10)	<0.001	1.04(0.90, 1.19)	<0.001

GC: Gynecological cancer; NC: Nasopharyngeal carcinoma; GUC: Genitourinary system cancer; GIC: Gastrointestinal cancer; BC: Breast cancer; N: number of studies included; OR: odds ratio; P_h_: p value for heterogeneity;

* OR with statistical significance;

a studies with more than 500 participants;

b studies with less than 500 participants;

### Evaluation of Heterogeneity

Heterogeneity between studies in each model is shown in Table 2 and Table 3. We investigated the source of heterogeneity by covariables, such as publication years, cancer types, ethnicities, source of controls, sample size and genotyping method. As for −607 C>A, although meta-regression analysis revealed that no covariables contributed to the heterogeneity across the studies in the overall result, sub-group analyses indicated that source of controls and sample size might be the main source of heterogeneity. As for −137 G>C, the meta-regression analysis revealed that cancer types (p = 0.039), but not other covariables contributed to the heterogeneity across studies in the overall result, which was in consistent with sub-group analyses.

### Sensitivity Analyses and Publication Bias

**Figure 4:**
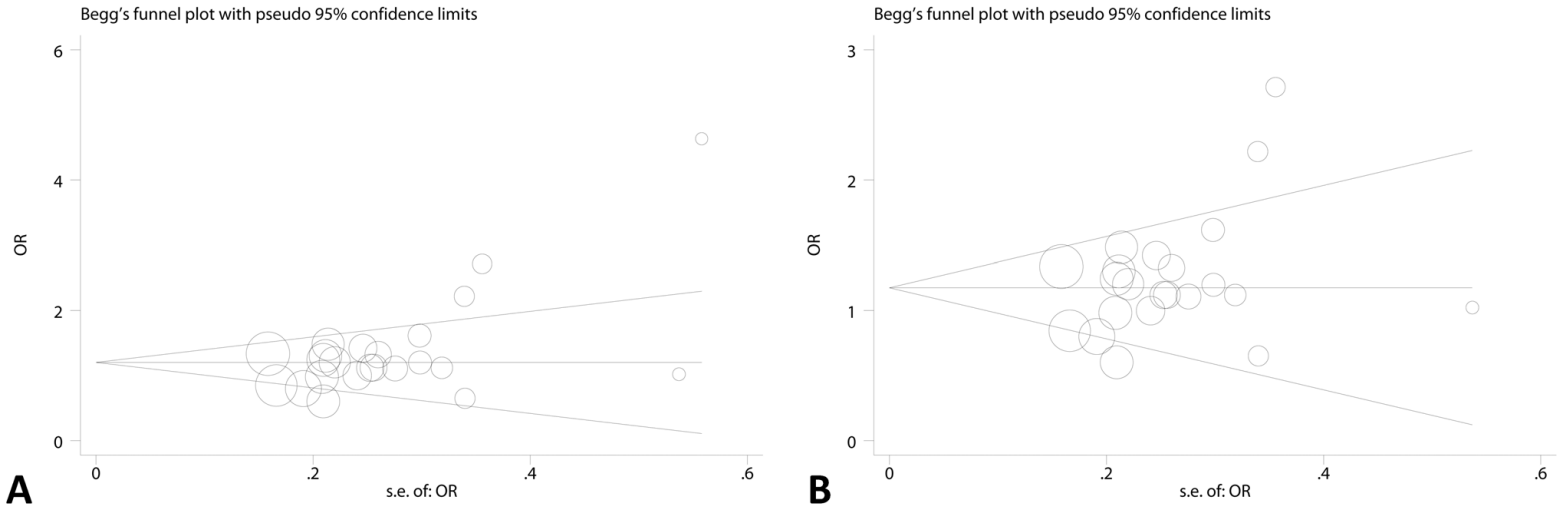
Funnel plot analysis to detect publication bias for −607 C>A. A: funnel plot of all 23 eligible studies on −607 C>A, Egger’s test p = 0.009. B: funnel plot of 22 studies on −607 C>A (Qi’s study was excluded), Egger’s test p = 0.103. The circles represent the weight of individual study.

**Figure 5:**
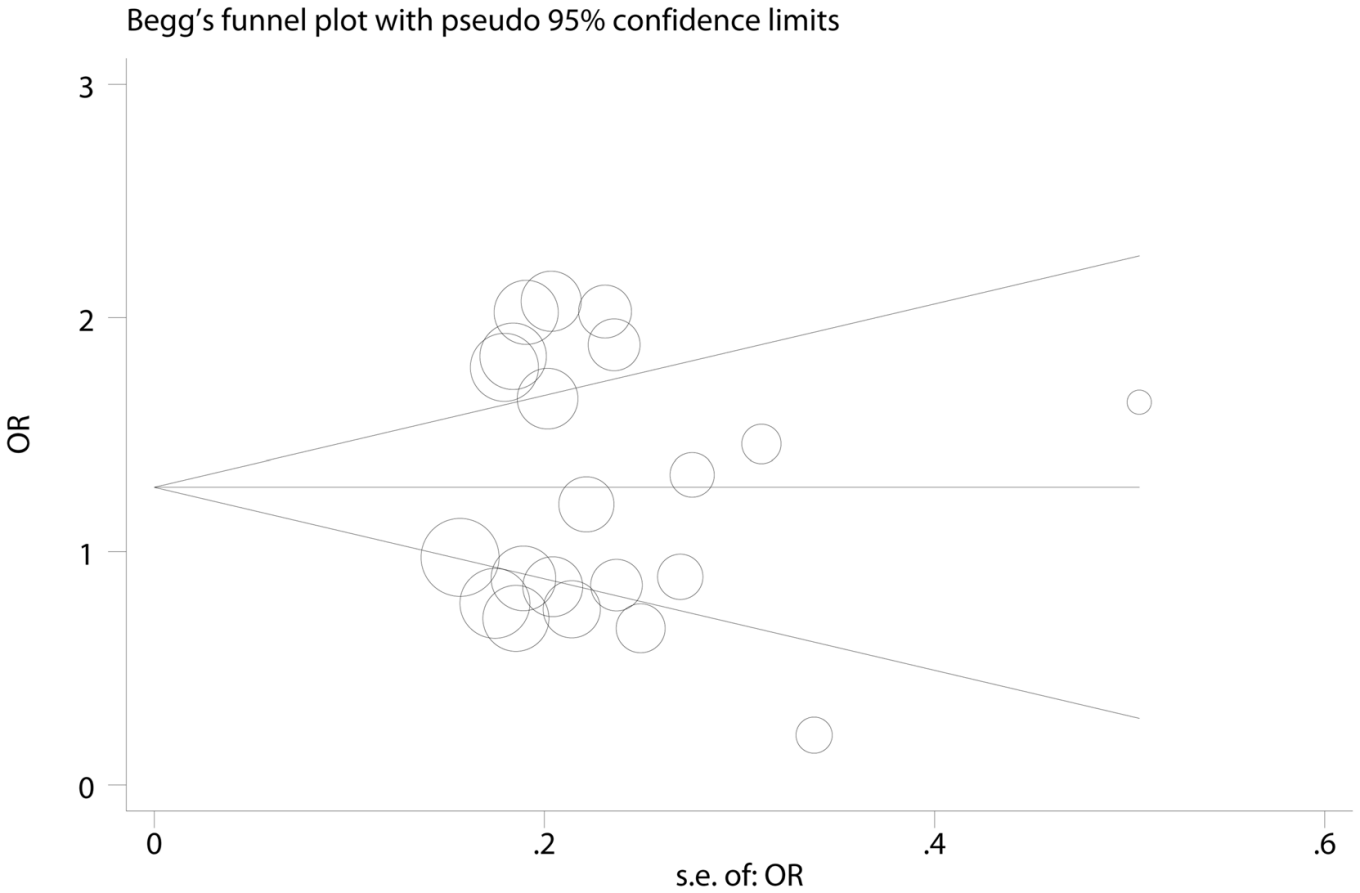
Funnel plot analysis to detect publication bias for −137 G>C. The circles represent the weight of individual study.

Sensitivity analysis was performed to estimate individual study’s influence on the pooled ORs by deleting one single study each time from pooled analysis, and the corresponding pooled ORs were not materially altered, suggesting stability of the meta-analyses (Figure S5 and Figure S6). Publication bias was assessed by Begg’s funnel plot and Egger’s test. Begg’s funnel plot was both roughly symmetrical for two polymorphisms (Figure 4.A and Figure 5). Egger’s test was then performed for statistical test, no publication bias was detected for −137 G>C (p = 0.842), but −607 C>A failed (p = 0.009). Further analysis revealed that the study reported by Qi and colleagues [15] was responsible for the asymmetry of funnel plot (Figure 4.A). When this study was deleted, there was no evidence of publication bias for −607 C>A (p = 0.103, Figure 4.B), while the pooled OR was marginally significant (OR = 1.14, 95% CI: 1.00, 1.30).

## Discussion

To our knowledge, this is the first meta-analysis to explore the association between IL-18 gene promoter polymorphisms (−607 C>A and −137 G>C) and cancer risk. In the present meta-analysis, 26 eligible studies including 4096 cases and 5222 controls, were identified and analyzed. We demonstrated that IL-18 gene promoter −607 C>A polymorphism was associated with a statistical increased risk of cancer susceptibility in the variant CA heterozygote and CA/AA genotype compared with the CC wild type homozygote, especially in nasopharyngeal carcinoma and gastrointestinal cancer, however, an opposite trend was found in genitourinary system cancer. Although no significant association for −137 G>C was observed with overall cancer risk, it is also worth noting that the association was significant in Asian populations, especially in nasopharyngeal carcinoma.

IL-18 is a 18.3kDa multifunctional cytokine and generally referred to as a member of the IL-1 family. IL-18 can enhance the production of IFN-γ by T cells and NK cells and augment the cytolytic activity of NK cells and cytotoxic T lymphocytes [41], [42]. It can also affect the differentiation of CD4+ and CD8+ T cells, and acts synergistically with other cytokines such as IL-12 to induce the production of IFN-γ and stimulate Th1 immune response [3]. Recently, many studies indicated that IL-18 might be closely related to the pathogenesis of tumors. The specific and non-specific anti-tumor effects were confirmed in IL-18 gene transfected dendritic cells and breast cancer cells [43], [44]. In addition, it has been reported that serum IL-18 level may be used as a marker for monitoring the clinical course of patients with some cancer types, including esophageal, breast and gastric cancer[45]–[47]. It has shown that the polymorphisms of IL-18 could influence gene activity and expression of IL-18 [9]. Together with the critical role of IL-18 in cancer immunity regulation, the polymorphisms of IL-18 would be related to cancer risks.

Among 23 eligible studies based on −607 C>A, we found a significant increased risk in the heterozygote comparison (CA vs. CC) and dominant model (CA/AA vs. CC) for nasopharyngeal carcinoma and gastrointestinal cancer, including colorectal cancer [13], [29], [37], esophageal carcinoma [14], [36] and stomach cancer [29], which was in consistent with our pooled analysis of overall cancer risk. However, a trend of reduced cancer risk was found in genitourinary system cancer, including prostate cancer [12], [40], renal cell carcinoma [33] and bladder cancer [39]. These results suggested that the variant CA and CA/AA genotypes of IL-18 gene promoter −607 C>A polymorphism were definitive associated with cancer susceptibility, especially in nasopharyngeal carcinoma and gastrointestinal cancer. In the sub-group analyses of ethnicities, no significant association except a trend of increased cancer risk was found in Asians and Caucasians. However, Jaiswal’s study was the only study which reported that the CA/AA genotype could be associated with reduced cancer risk in Asians [39]. The contrary individual result might be attributed to the discrepancy between bladder and other cancers. So we found that cancer types greatly affected the association between IL-18 gene promoter −607 C>A polymorphism and cancer risk, but ethnicities failed.

Among 21 eligible studies based on −137 G>C, carriers of the variant C allele were only reported with a significantly increased cancer risk compared with those of G allele in nasopharyngeal carcinoma [10], [11], [31], [38]. In dominant model, although many single studies suggested −137 G>C polymorphism significantly contributed to the susceptibility of other cancer types, including cervical [26], prostate [12], bladder [39], esophageal [14] and colorectal cancer [37], the pooled ORs failed to confirm the association in each corresponding group classified by cancer types. Furthermore, Monroy and colleagues found significantly reduced cancer risk with GC/CC genotype in hodgkin disease [34]. This is the only negative result among all eligible studies. In the sub-group analysis of cancer types, no significant association was found except for four models of nasopharyngeal carcinoma. Moreover, the −607A/−137C and −607C/−137C haplotypes were significantly associated with the risk of nasopharyngeal carcinoma. Notably, both haplotypes included a variant −137C allele. This finding suggests that the IL-18 −137 G>C polymorphism could be used as a genetic susceptibility marker of nasopharyngeal carcinoma. But for the four studies of genitourinary system cancer, two of them found significant increased risk with C variant allele carriers [12], [39], while the other two of them found a trend of reduced cancer risk in contrast [33], [40]. Likewise, no significant association was detected in gastrointestinal cancer, while two of them found significant increased cancer risk [14], [37], the other three studies found a trend of reduced cancer risk [29], [36].This discrepancy may be explained by the reason that the detailed pathology types were different. Moreover, ethnicity might be also an important reason, because the studies which reported increased cancer risk were all most carried out in Asians. We also found the association between the −137 G>C and cancer risk was significant in Asians, but a trend of reduced cancer risk was found in Caucasians. The differences might be explained by genetic diversities, such as different risk factors in life styles, and various of environmental exposure. Additionally, in the sub-group analysis of the source of controls, the positive result was only observed in hospital-based studies, but not in population-based studies. However, the hospital-based controls might not represent of the general population, thus there was a low chance of selection bias.

As for the aforementioned publication bias detected by Egger’ test (CA/AA vs. CC) for −607 C>A, Qi’s study [15]was responsible for the bias. However, when we excluded it, the pooled OR was marginally significant (OR = 1.14, 95% CI: 1.00, 1.30). Thus we speculated that the publication bias we detected might contribute to publishing positive results. Therefore, it is expected that more studies are required to confirm the pooled OR in this meta-analysis, and the funnel plot will be more symmetrical and no publication bias will be detected.

For heterogeneity, we found that sample size was the main source of heterogeneity for both polymorphisms. The studies with small sample size may contribute to a small-study effect, in which effects reported are larger, and lead to between studies variance. However, this kind of heterogeneity is hard to exclude, because recruitment of enough cases with specific cancer type is difficult.

In this meta-analysis, we included 4096 cases and 5222 controls, which can provide enough statistical power and strengthen the reliability of our results. In addition, several limitations should be considered: First, detailed individual data was not available, and a more precise analysis should be conducted on other covariates such as age, sex, and environmental factors. Secondly, the sample size was relatively small for some sub-group analyses. Thirdly, a tiny publication bias for −607 C>A excited in this meta-analysis.

In conclusion, we demonstrate that IL-18 gene promoter −607 C>A polymorphism is significantly associated with overall cancer risk, especially in nasopharyngeal carcinoma and gastrointestinal cancer; and the −137 G>C polymorphism is associated with increased overall cancer risk in Asian populations and also significantly increase the risk of nasopharyngeal carcinoma. Future large-scale studies are required to validate the current findings.

## Supporting Information

Figure S1 **Forest plot of −607 C>A heterozygote comparison for overall comparison by source of controls (CA vs. CC).**
(TIF)

Figure S2 **Forest plot of −607 C>A dominant model for overall comparison by sample size (CA/AA vs. CC).**
(TIF)

Figure S3 **Forest plot of −137 G>C dominant model for overall comparison by ethnicities (GC/CC vs. GG).**
(TIF)

Figure S4 **Forest plot of −137 G>C dominant model for overall comparison by source of controls (GC/CC vs. GG).**
(TIF)

Figure S5 **Sensitivity Analyses for −607 C>A.** The pooled odds ratios were calculated by omitting each data set at a time.
(TIF)

Figure S6 **Sensitivity Analyses for −137 G>C.** The pooled odds ratios were calculated by omitting each data set at a time.
(TIF)

Checklist S1 **PRISMA checklist.**
(DOC)
