# Supplementary figures and images for: Association of Interleukin-18 Gene Promoter −607 C>A and −137G>C Polymorphisms with Cancer Risk: A Meta-Analysis of 26 Studies
Source: PLoS One. 2013 Sep 16;8(9):e73671. doi: 10.1371/journal.pone.0073671 (PMC3774799; doi:10.1371/journal.pone.0073671)

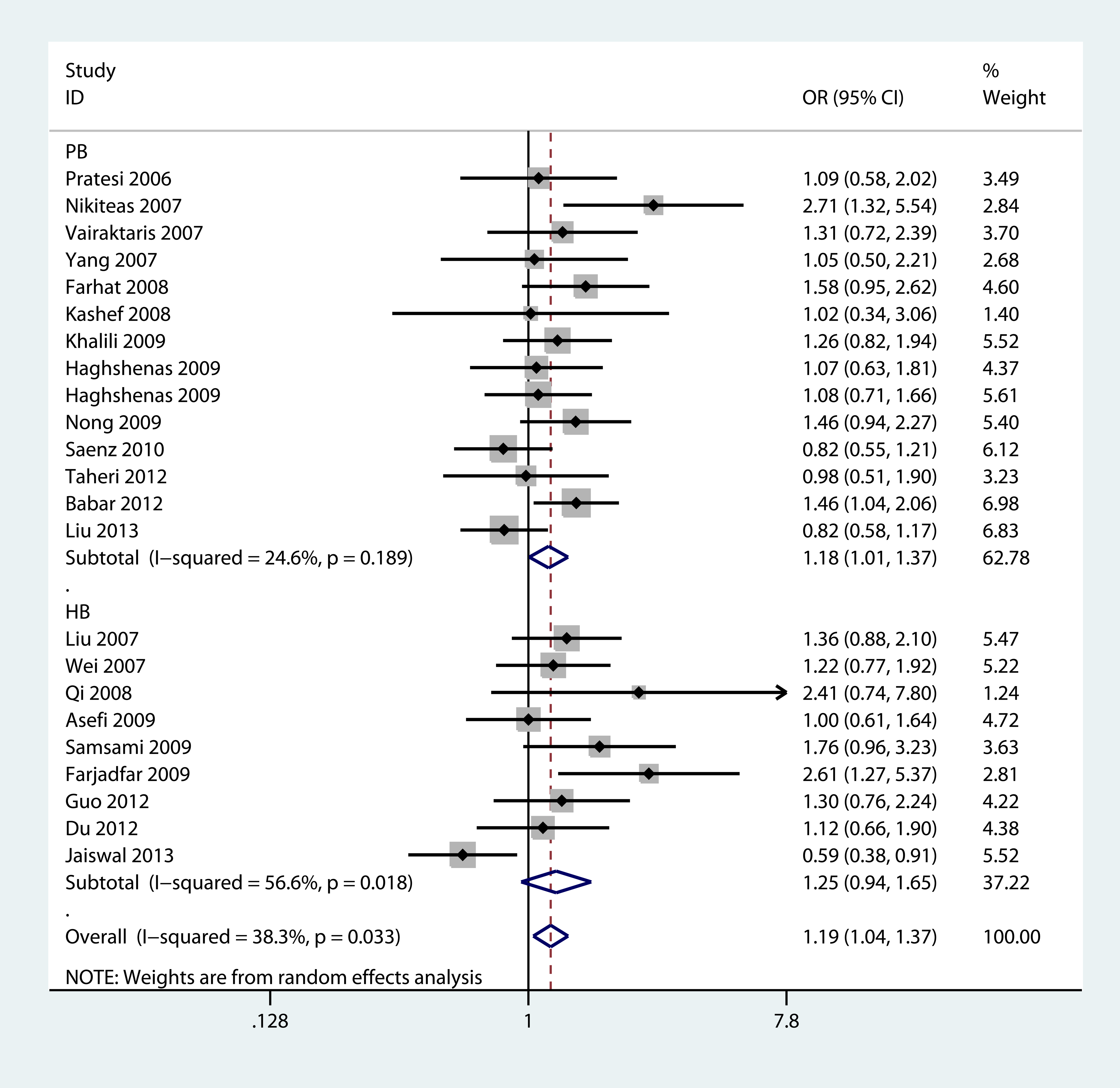

Supplement: Figure S1 — Forest plot of −607 C>A heterozygote comparison for overall comparison by source of controls (CA vs. CC). (TIF) [file pone.0073671.s001.tif]

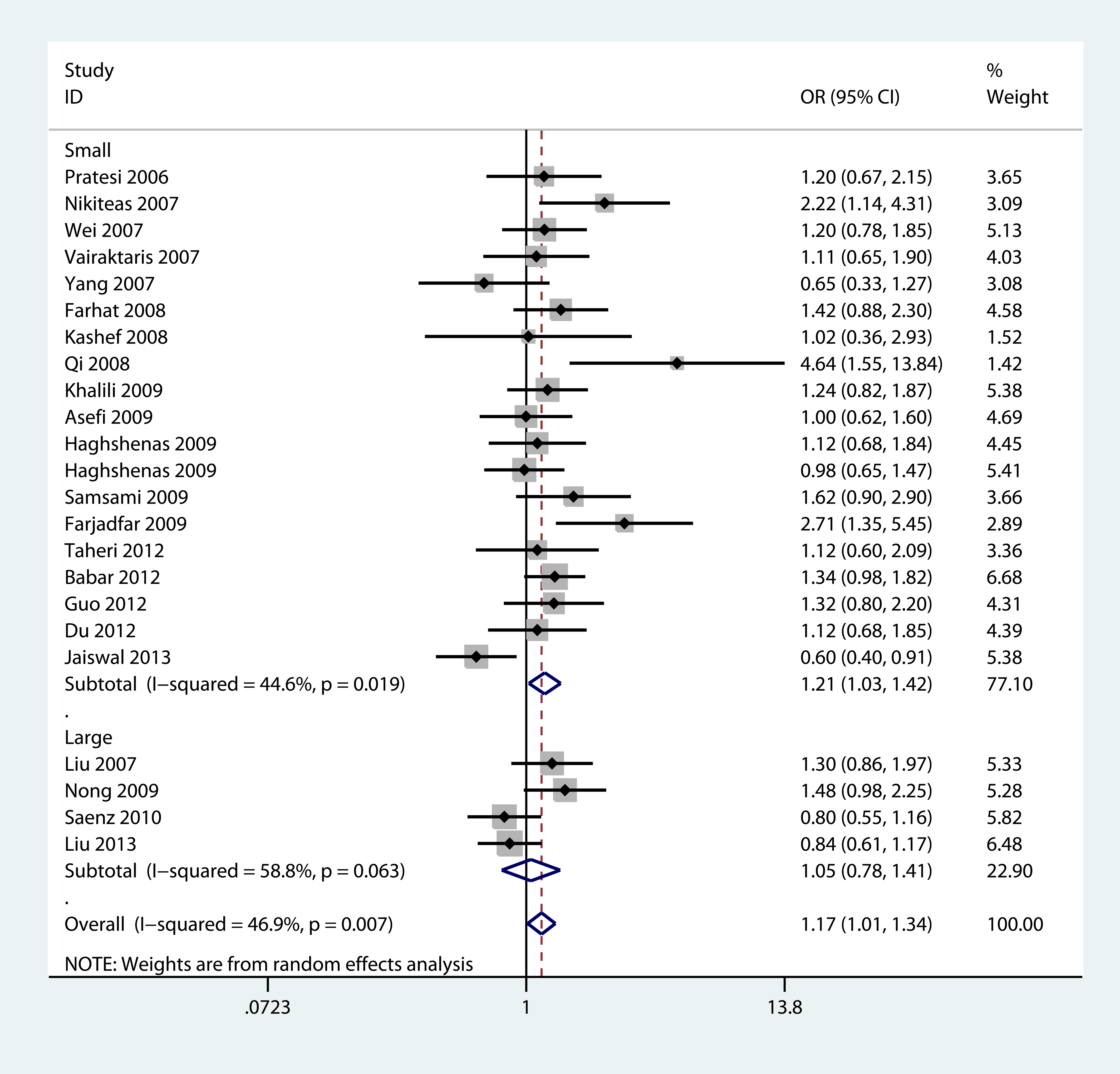

Supplement: Figure S2 — Forest plot of −607 C>A dominant model for overall comparison by sample size (CA/AA vs. CC). (TIF) [file pone.0073671.s002.tif]

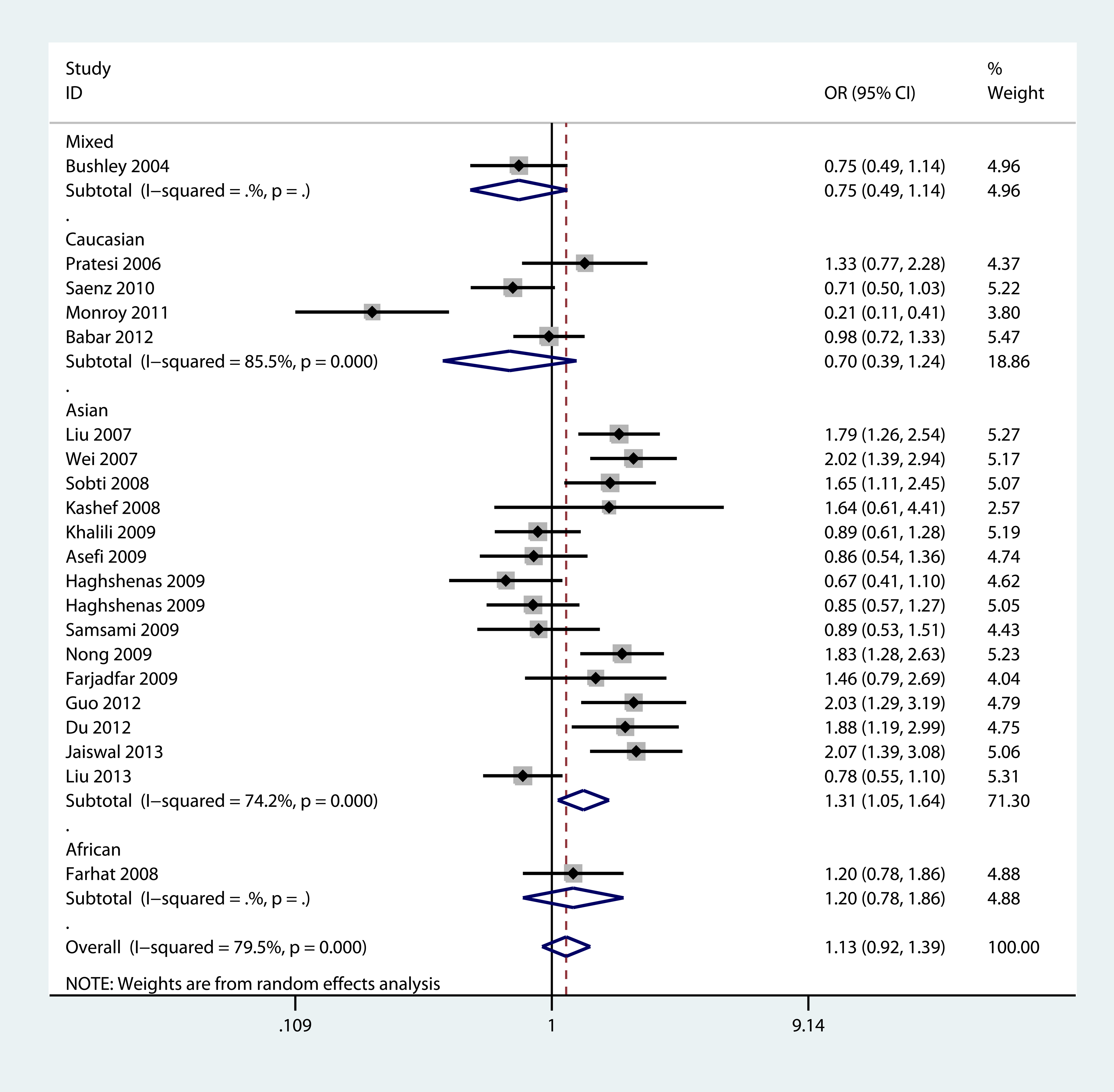

Supplement: Figure S3 — Forest plot of −137 G>C dominant model for overall comparison by ethnicities (GC/CC vs. GG). (TIF) [file pone.0073671.s003.tif]

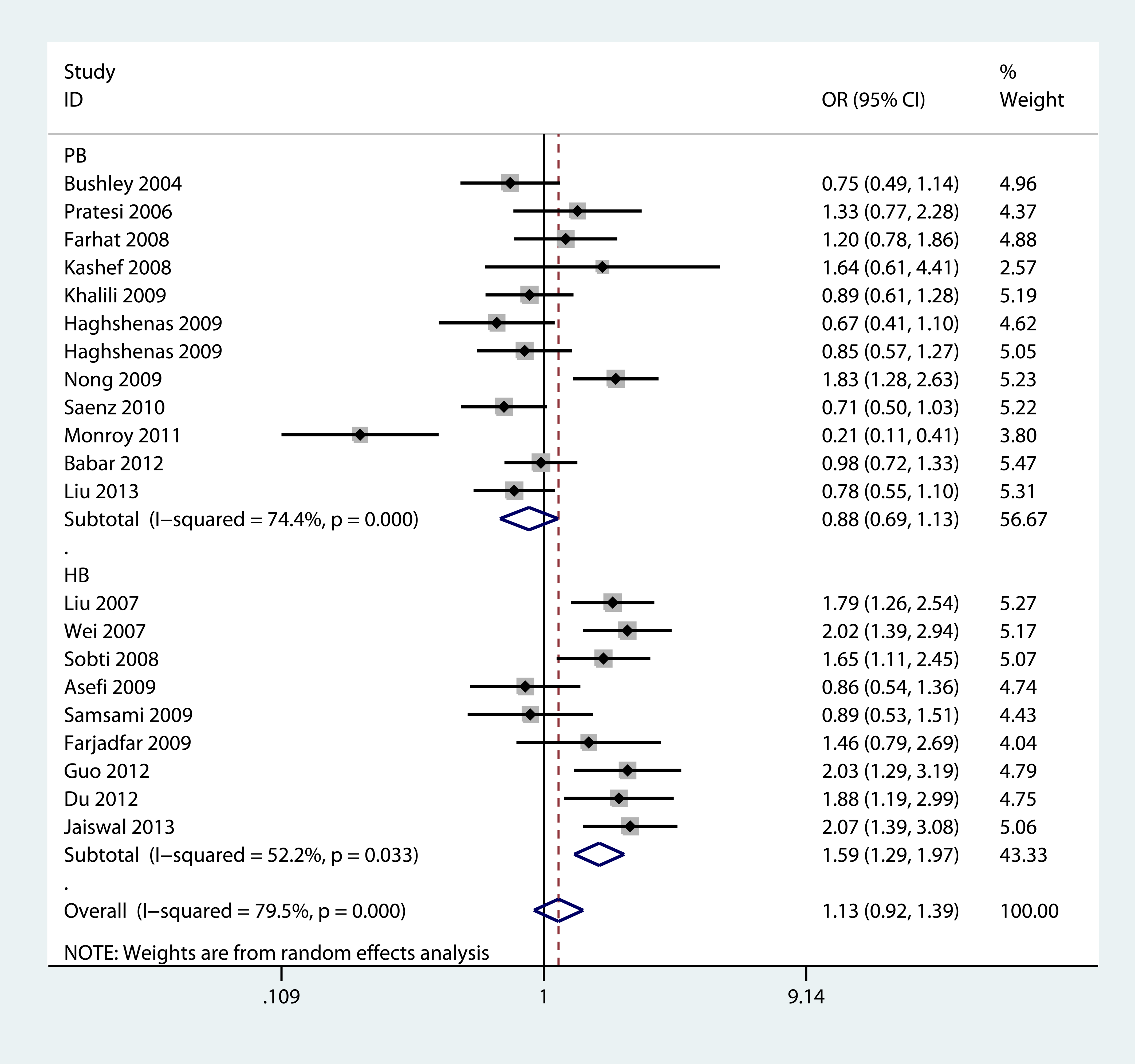

Supplement: Figure S4 — Forest plot of −137 G>C dominant model for overall comparison by source of controls (GC/CC vs. GG). (TIF) [file pone.0073671.s004.tif]

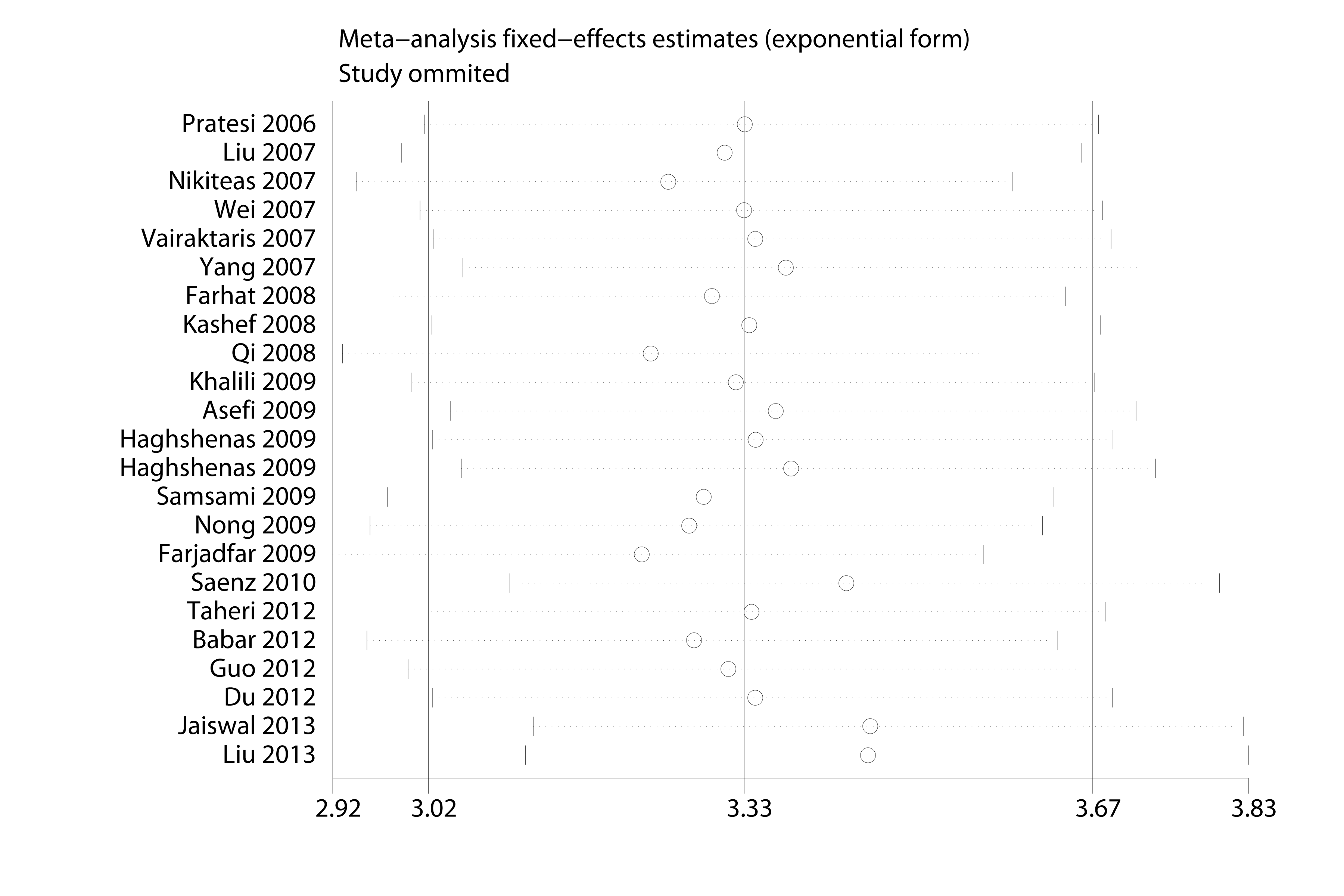

Supplement: Figure S5 — Sensitivity Analyses for −607 C>A. The pooled odds ratios were calculated by omitting each data set at a time. (TIF) [file pone.0073671.s005.tif]

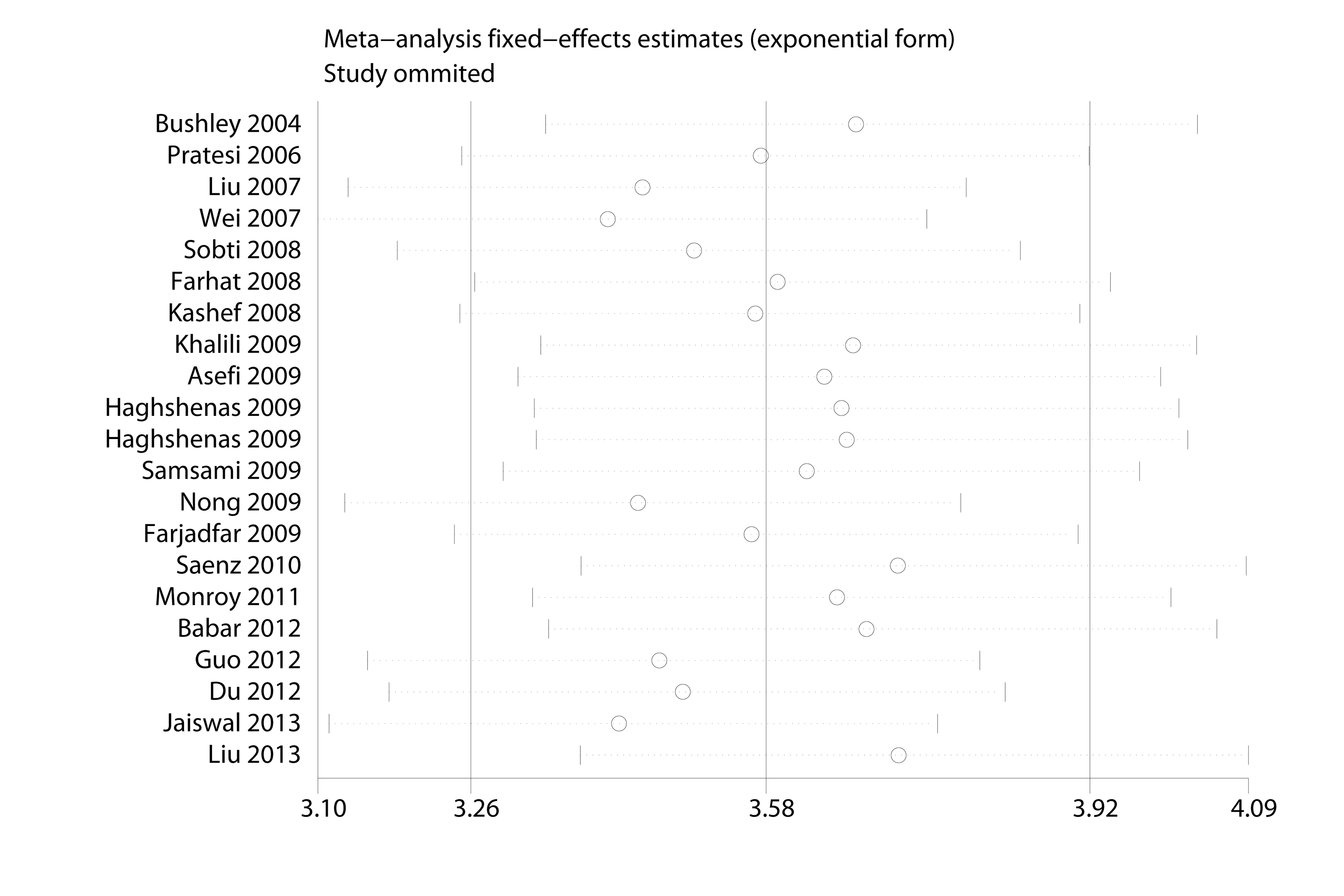

Supplement: Figure S6 — Sensitivity Analyses for −137 G>C. The pooled odds ratios were calculated by omitting each data set at a time. (TIF) [file pone.0073671.s006.tif]
